# Early Changes in the White Matter Microstructure and Connectome Underlie Cognitive Deficit and Depression Symptoms After Mild Traumatic Brain Injury

**DOI:** 10.3389/fneur.2022.880902

**Published:** 2022-06-30

**Authors:** Wenjing Huang, Wanjun Hu, Pengfei Zhang, Jun Wang, Yanli Jiang, Laiyang Ma, Yu Zheng, Jing Zhang

**Affiliations:** ^1^Department of Magnetic Resonance, Lanzhou University Second Hospital, Lanzhou, China; ^2^Second Clinical School, Lanzhou University, Lanzhou, China; ^3^Gansu Province Clinical Research Center for Functional and Molecular Imaging, Lanzhou, China

**Keywords:** brain injury, DTI, WM microstructure, cognitive deficits, depression symptoms

## Abstract

Cognitive and emotional impairments are frequent among patients with mild traumatic brain injury (mTBI) and may reflect alterations in the brain structural properties. The relationship between microstructural changes and cognitive and emotional deficits remains unclear in patients with mTBI at the acute stage. The purpose of this study was to analyze the alterations in white matter microstructure and connectome of patients with mTBI within 7 days after injury and investigate whether they are related to the clinical questionnaires. A total of 79 subjects (42 mTBI and 37 healthy controls) underwent neuropsychological assessment and diffusion-tensor MRI scan. The microstructure and connectome of white matter were characterized by tract-based spatial statistics (TBSSs) and graph theory approaches, respectively. Mini-mental state examination (MMSE) and self-rating depression scale (SDS) were used to evaluate the cognitive function and depressive symptoms of all the subjects. Patients with mTBI revealed early increases of fractional anisotropy in most areas compared with the healthy controls. Graph theory analyses showed that patients with mTBI had increased nodal shortest path length, along with decreased nodal degree centrality and nodal efficiency, mainly located in the bilateral temporal lobe and right middle occipital gyrus. Moreover, lower nodal shortest path length and higher nodal efficiency of the right middle occipital gyrus were associated with higher SDS scores. Significantly, the strength of the rich club connection in the mTBI group decreased and was associated with the MMSE. Our study demonstrated that the neuroanatomical alterations of mTBI in the acute stage might be an initial step of damage leading to cognitive deficits and depression symptoms, and arguably, these occur due to distinct mechanisms.

## Introduction

Mild traumatic brain injury (mTBI), commonly known as concussion, is a significant public health problem. Every year, an estimated 42 million people worldwide suffer from mTBI (1), accounting for approximately 70–90% of all traumatic brain injuries (TBIs). mTBI causes various symptoms, most notably headaches, fatigue, depression, anxiety, and impaired cognitive function, termed as post-concussion syndrome. These symptoms may resolve in most individuals within 3 months post-injury. Nevertheless, mTBI is more likely a progressive injury than a static event (2). Some patients (20–30%) go on to develop persistent physical (headache, dizziness, and fatigue) (3), emotional (irritability, depression, anxiety, and posttraumatic stress) (4), and cognitive deficits (5). Diagnosing and predicting mental and emotional deficits early after an injury is essential to avoid persistent symptoms that may pose a barrier to full recovery.

Diffusion tensor imaging (DTI) and related techniques are sensitive for assessing white matter (WM) microstructure changes after injury, but the results are heterogeneous (6). Changes in WM structural abnormalities have been observed in both animals and humans following brain injury (7, 8). Based on a rodent model of mTBI, it was found that radial diffusivity (RD) and axial diffusivity (AD) decreased one week after injury. Simultaneously, fractional anisotropy (FA) increased and returned to baseline values after 2 weeks (7). In patients with mTBI, DTI found higher FA and lower RD in the corpus callosum when measured 2–20 days after mTBI and higher FA in the genu of the corpus callosum after follow-up (8). Another study showed that patients with persistent post-concussion symptoms had lower FA and higher RD values than patients without symptoms when examined with DTI within 72 h post-injury (9). DTI studies on mTBI have also reported WM structural disruption associated with cognitive and affective impairments after mTBI. For instance, the patient's cognitive information processing speed was associated with FA values recovered in the left anterior limb of the internal capsule (10). Damage to the frontal interhemispheric and thalamic projection tracts can be used to predict processing speed performance in mTBI (11). Damage to the left superior longitudinal fasciculus and right anterior thalamic radiation is closely related to pain emotion regulation (12).

Region-of-interest (ROI)-based research has provided insights into the mechanisms that underlie mTBI-related cognitive and emotional deficits (13, 14). However, the human brain is an integrated network for processing higher-order cognition and emotion. Network theory, especially, the WM structural connectome, provides a way to simplify complex systems into more straightforward representations to investigate brain organization and capture the topological characteristics of the network (15, 16). To date, many studies have used theoretical graph techniques to explore WM structural network alterations in TBI. One study found that patients demonstrated increased shortest path length and decreased the global efficiency of the structural network, which was associated with lower scores on switching tasks (17). Reduced network efficiency is associated with the rupture of long-range WM connections due to axonal injury, which impairs information transfer across distal brain regions (18) and leads to cognitive deficits (19). Although some studies have found TBI-induced structural brain network alterations, others have reported no significant alterations in the global network metrics (20, 21). It is unknown whether patients with mTBI have global and local topologic alterations in the connectome.

In addition, previous studies primarily focused on moderate and severe brain injury or the chronic stages of mTBI (22, 23). Few studies have examined acute brain changes (24). In contrast, acute brain injury is a golden time for mTBI treatment (25). The neuroanatomical abnormalities associated with acute mTBI have not yet been thoroughly investigated. Similarly, the precise position and topological way of WM connectivity are disrupted, and consequently, how much cognitive, and emotional impairments are affected remains unclear. Therefore, this study aimed to assess microstructural changes in the acute phase of mTBI, employ graph theory to explore alterations in the WM connectome, and further explore the potential mechanism of cognitive decline and depressive symptoms at the acute stage of mTBI. We hypothesized that mTBI would change the structural network properties, which might be related to mental/emotional impairments in the acute phase.

## Materials and Methods

### Subjects

In total, forty-eight patients and forty age-, sex-, and education-matched healthy controls (HCs) were recruited from the Lanzhou University Second Hospital. Patients with mTBI were seen at the Department of Emergency and Neurosurgery within 7 days after injury. Most of them were outpatients and emergency patients. The inclusion criteria were as follows: (i) the patient (18–60 years and right-handed) had suffered a brain injury within 7 days; (ii) met at least one of the diagnostic criteria of the American Congress of Rehabilitation Medicine for mTBI (26); (iii) no substantial trauma could be found on brain computed tomography and routine MRI; and (iv) no MRI contraindications. The exclusion criteria were as follows: (i) severe compound injury and multiple injuries; (ii) history of previous TBI or neuropsychological, psychiatric, or neurological disorders; and (iii) history of drug or alcohol abuse. The healthy subjects were carefully screened according to the same exclusion criteria applied to the patient group.

### Neuropsychological Assessment

All the subjects underwent the same neuropsychological tests which are commonly affected domains after mTBI. (i) Mini-mental state examination (MMSE) to assess cognitive function. (ii) Self-rating depression scale (SDS) to evaluate adults with depressive symptoms in the last week. (iii) The mTBI group completed the Rivermead post-concussion syndrome questionnaire (RPQ), which provides a symptom questionnaire commonly reported after mTBI to reflect the level of post-concussive symptoms (27).

### MRI Data Acquisition

Magnetic resonance imaging data were acquired on a 3T Siemens Verio scanner equipped with an 8-channel phased-array head radiofrequency coil. (i) T2-weighted fluid-attenuated inversion recovery images (FLAIR; TE = 94 ms, TR = 7,000 ms, TI = 2,215.2 ms; FOV: 230 × 230 mm; matrix: 256 × 256; 18 slices) to eliminate cerebral contusion and laceration, subarachnoid hemorrhage, and cerebral softening lesion. (ii) Sagittal three-dimensional T1-weighted images (3D-T1WI) were acquired using turbo spin-echo sequence (TI = 900 ms; flip angle = 12°; FOV: 256 × 256 mm; matrix: 256 × 256; 192 slices). (iii) The diffusion tensor images (DTIs) were acquired using a single-shot echo-planar sequence (TE = 61 ms; TR = 7,100 ms; flip angle = 90°; FOV: 256 × 256 mm; matrix: 128 × 128; 50 slices; 64 diffusion directions, *b* value of 1,000 and 0 s/mm^2^).

### Tract-Based Spatial Statistics (TBSSs)

The preprocessing and analysis steps based on the TBSS method (28) were performed using the FMRI Software Library (FSL, http://fsl.fmrib.ox.ac.uk/fsl). First, diffusion tensor images were corrected for the eddy current distortions and head motion, and the scalp, skull, and other non-brain tissues were removed from the images. Next, we automatically estimated all the WM tracts using the TBSS method. The FA data of all the subjects were then aligned to the FMRIB Diffusion Toolbox module. A mean FA image was created, and the threshold (0.2) was designed to create a mean FA skeleton. The FA, MD, AD, and RD data aligned for each subject were projected onto this skeleton. The randomization of FSL for nonparametric permutation, 5,000 permutation tests, and threshold-free cluster enhancement and familywise error (FWE) were used for multiple comparisons and corrections. Statistically significant between-group differences were identified using the Johns Hopkins University (JHU) WM tractography atlas.

### WM Structural Network Construction and Analyses

The nodes of the brain structural network came from the automated anatomical labeling template, which divides the brain into 90 brain regions (29). MRtrix3's tckgen with constrained spherical deconvolution and anatomically constrained tractography was used for probabilistic tracking of whole-brain WM fibers (step size = 1 mm, angle threshold = 45°, the threshold of FOD = 0.05, 1 million streamlines) (30). The weight of the edges between nodes is defined as the number of streamlines connecting the nodes in the two brain regions. Edges with streamlines <15 were deleted to eliminate potential spurious connections.

Based on MatlabR2013b, GRETNA (http://www.nitrc.org/projects/Gretna/) software was used to generate graph theory metrics (31). Network efficiency was examined at both the global and local levels. For the global network theory analyses, the theoretical network measures calculated were the clustering coefficient (Cp, reflecting the tendency of nodes to cluster together), characteristic path length (Lp, network average of the shortest path lengths), small-worldness (reflecting the ability of integration and segregation in the network), global efficiency (Eg, calculated by taking the mean inverse shortest path length in the network), local efficiency (E_local_, efficiency of connections between neighbors of a node), and hierarchy (revealing an organizational structure with a high degree of clustering). For the local network theory analyses, the central degree (Dc, the number of edges attached to each node), nodal efficiency (Ne, reflects the efficiency of information dissemination between nodes), and nodal shortest path length (NLp, the size of the shortest path between nodes) were calculated.

A nonparametric one-tailed sign test was used to determine group networks and hub areas. The entire network can be divided into the hub and non-hub nodes. The node of the hub region is higher than the intermediate node with at least one SD, which has the shortest path connection with other nodes and high centrality and efficient information transmission capability (32). Correspondingly, three subnetworks were defined: rich club (connecting hub nodes only), feeder (connecting hub and non-hub nodes), and local connection (connecting non-hub nodes only). The connection strength of each subnetwork (number of streamlines) was calculated for the statistical analyses.

### Statistical Analyses

The demographic and neuropsychological data of the mTBI and HC groups were analyzed using SPSS version 22.0, using independent two-sample *t*-tests for continuous variables with a normal distribution (age, MMSE, and SDS) and chi-square tests for sex. Given the non-normal distributions of education years, the Mann–Whitney *U* tests were utilized. Multiple comparisons were corrected using false discovery rate (FDR) correction for local network properties. Nonparametric permutation tests (10,000 times) were used to test for group differences in the three subnetworks of connectivity strength, other network topology metrics, and nodal efficiency with age, sex, and years of education as covariates. Network-based Statistics (NBS) (33) were used to evaluate the abnormalities of anatomical connections in the network between groups. The network connections of each subject were tested using a permutation test (10,000 times) controlled for FWE correction, and the threshold was set to a *p-*value of <0.05. In the mTBI group, clinical assessments were correlated with graph theory outcomes using Spearman's correlation analysis.

## Results

### Demographic and Clinical Characteristics

In total, six patients and three healthy subjects were excluded due to poor-scan quality from motion artifacts. The final sample consisted of 79 participants, including 42 patients with acute mTBI and 37 HCs (Table 1). The two groups did not differ in terms of age, gender, or educational distribution. Patients with mTBI had varying degrees of headaches, fatigue, insomnia, depression, and abnormally increased RPQ scores. Compared with the HCs, patients with mTBI performed worse on the MMSE test (*p* < 0.001) and showed a significantly higher SDS score (*p* < 0.001).

**Table 1:** Demographic and neuropsychological assessment in patients with mTBI and HCs.

	**mTBI (*n* = 42)**	**HCs (*n* = 37)**	***p*-value**
Age, years, mean (SD)^a^	34.17 (11.30)	35.46 (10.72)	0.627
Gender, Male, *n* (%)^b^	23 (54.76)	19 (51.35)	0.762
Education, years, median (IQR)^c^	8 (10)	8 (9.5)	0.660
Injury-to-imaging interval, hours, median (IQR)	72 (78)	–	–
Loss of consciousness (LOC), *n* (%)	15 (35.70)	–	–
Time to resumption of LOC, minutes, median (IQR)^d^	5 (28)	–	–
Posttraumatic amnesia (PTA), *n* (%)	12 (28.60)	–	–
Time to resumption of PTA, hours, median (IQR)^e^	2.25 (3)	–	–
Microbleeds, *n* (%)	14 (33.3)	–	–
**Mechanism of injury**, ***n*** **(%)**
Vehicle accidents	23 (54.76)	–	–
Violence or assault	10 (23.81)	–	–
Athletic collisions	7 (16.67)	–	–
Others (incidental fall; falling object)	2 (4.76)	–	–
GCS, mean (SD)	14.52 (0.77)	–	–
RPQ, mean (SD)	16.40 (7.86)	–	–
MMSE, mean (SD)^a^	23.98 (6.19)	28.65 (1.95)	<0.001
SDS, mean (SD)^a^	46.67 (11.99)	32.56 (4.75)	<0.001

*mTBI, mild traumatic brain injury; HCs, healthy controls; GCS, Glasgow Coma Score; RPQ, Rivermead post-concussion syndromes questionnaire; MMSE, Mini-Mental State Examination; SDS, Self-rating Depression Scale; SD, standard deviation; IQR, interquartile range*.

a *The independent two-sample t-test was used for the continuous variables*.

b *The χ^2^ test was used for sex*.

c *The Mann–Whitney U test was used for education*.

d *n = 15*.

e *n = 12*.

### TBSS Results

**Figure 1:**
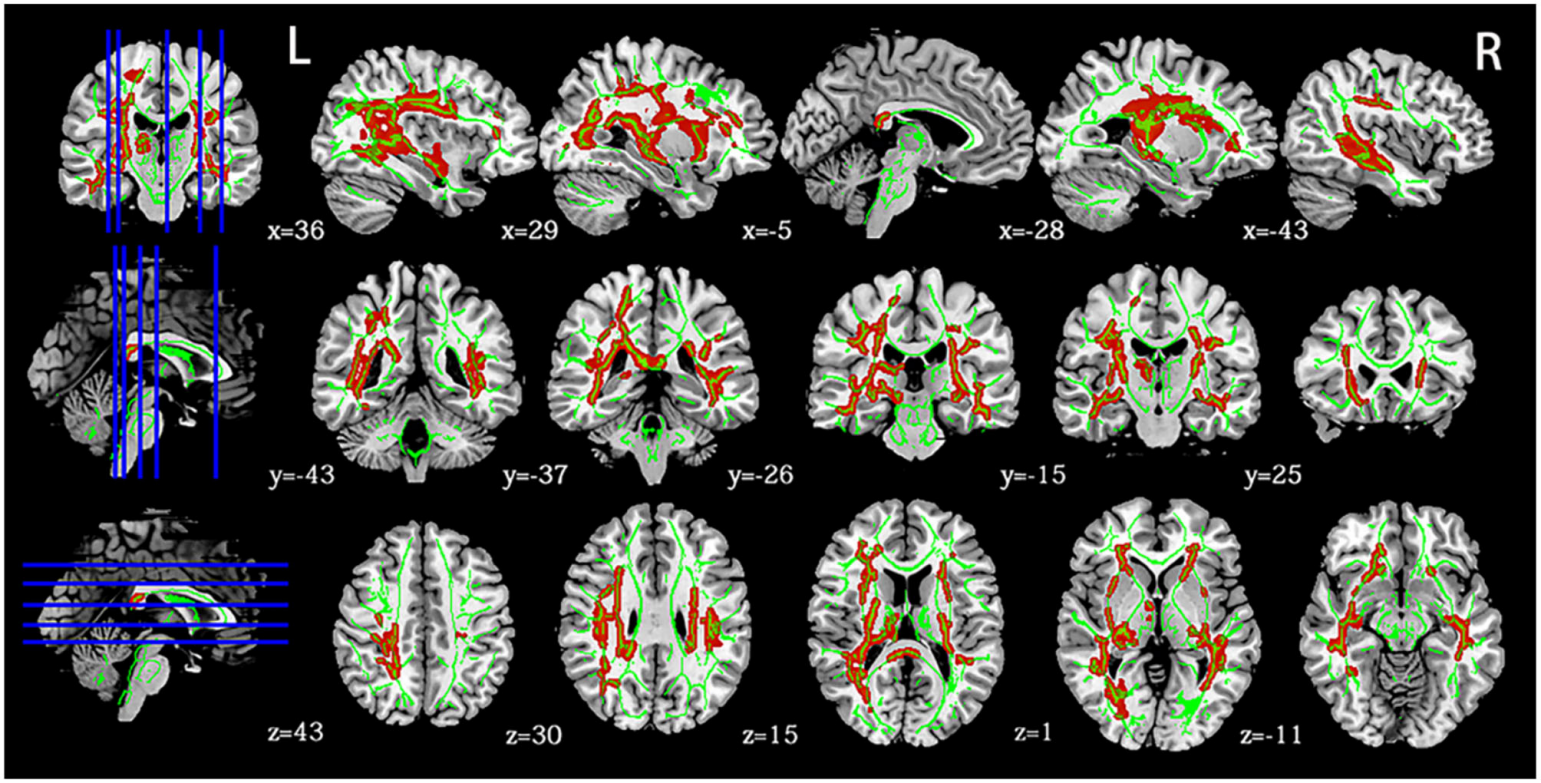
Panel highlights regions with higher FA in mTBI at the acute stage than HCs (*p* < 0.05, FWE). Green indicates a white matter skeleton. Red indicates voxels with higher FA using the “tbss_fill” script. L, left, R, right.

Voxel-wise analysis using TBSS showed that the mTBI group had a more significant number of clusters with increased FA than the HCs (corrected *p* < 0.05). These clusters belonged to seven tracts of the JHU atlas, including the bilateral inferior frontal-occipital fasciculus, superior longitudinal fasciculus, left uncinate fasciculus, left anterior thalamic radiation, and right inferior longitudinal fasciculus (Figure 1). Significantly, different voxels and their corresponding cluster sizes are shown in Table 2. The affected tracts were located predominantly in the left uncinate fasciculus and inferior frontal-occipital fasciculus. Voxel-wise analysis showed no significant changes in MD, AD, or RD between the two groups. There was no statistical correlation between FA values and neuropsychological indices in the different areas.

**Table 2:** The anatomical distribution with increased FA in acute mTBI group.

**Cluster**	**Voxels**	**min *P***	**Anatomical regions**
1	10,434	0.014	Uncinate fasciculus, Inferior fronto-occipital fasciculus (left)
2	3,534	0.027	Inferior fronto-occipital fasciculus, Superior longitudinal fasciculus, Uncinate fasciculus (left)
3	538	0.044	Inferior fronto-occipital fasciculus, Anterior thalamic radiation, Uncinate fasciculus (left)
4	34	0.049	Inferior fronto-occipital fasciculus, Superior longitudinal fasciculus, Inferior longitudinal fasciculus (right)

### Graph Theory Analyses Results

**Figure 2:**
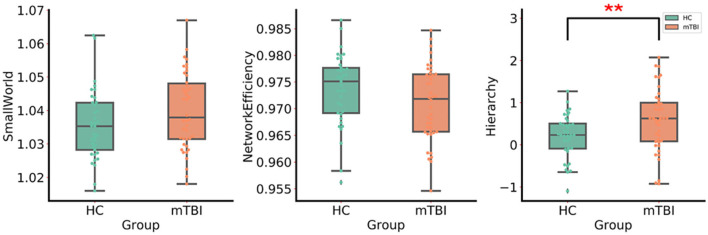
The group differences in the global network metrics of WM structural networks. ** Indicates *p* < 0.05. mTBI, mild traumatic brain injury; HC, healthy control.

Small-world organizations of the WM network were detected in both the mTBI and HC groups. An increase in the hierarchy (*p* < 0.05) of patients with acute mTBI was found when compared with the HCs (Figure 2). There was no significant difference in the between-group comparison of other global topological properties, including small-worldness, network efficiency, clustering coefficient, and characteristic path length.

**Figure 3:**
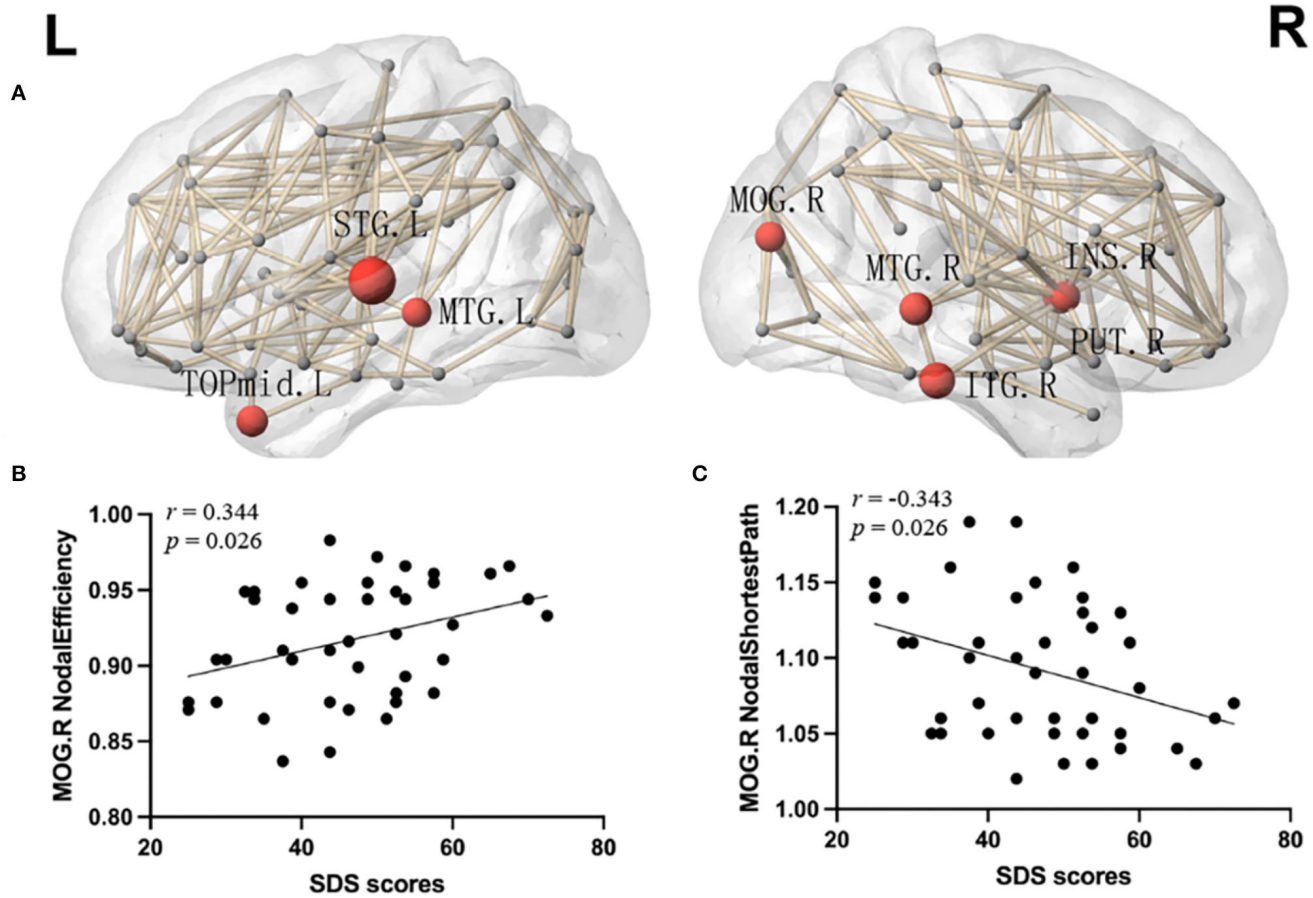
**(A)** Network hubs were colored in red to indicate significantly lower nodal efficiency and the node sizes indicate the significance (*p* < 0.05, FDR). INS, insular; MOG, middle occipital gyrus; PUT, putamen; STG, superior temporal gyrus; MTG, middle temporal gyrus; TPOmid, temporal pole of middle temporal gyrus; ITG, inferior temporal gyrus; L, left; R, right. **(B)** Correlation analyses between the SDS score and nodal efficiency of the right middle occipital gyrus. **(C)** Correlation analyses between the SDS score and nodal shortest path in the right middle occipital gyrus. SDS, Self-rating Depression Scale.

More significant results were found in the local networks. Compared with the HCs, the mTBI group showed an increased nodal shortest path length in the bilateral middle temporal gyrus, right insula, right middle occipital gyrus, right putamen, right inferior temporal gyrus, left superior temporal gyrus, left temporal pole of the middle temporal gyrus, and left angular gyrus. Except for the left angular gyrus, nodal efficiency (Figure 3A), and nodal degree centrality in the above brain regions were decreased in the mTBI group (*p* < 0.05, FDR). Moreover, Spearman's correlation analysis showed a positive correlation between the total SDS scores and nodal efficiency in the right middle occipital gyrus of patients with acute mTBI (*r* = 0.344, *p* = 0.026) (Figure 3B). In addition, a negative correlation between the total SDS scores and the shortest nodal path length in the right middle occipital gyrus of patients was detected (*r* = −0.343, *p* = 0.026) (Figure 3C). There was no correlation between local network properties of the right middle occipital gyrus and SDS scores in the healthy controls.

### Connected Edge Analyses Results

**Figure 4:**
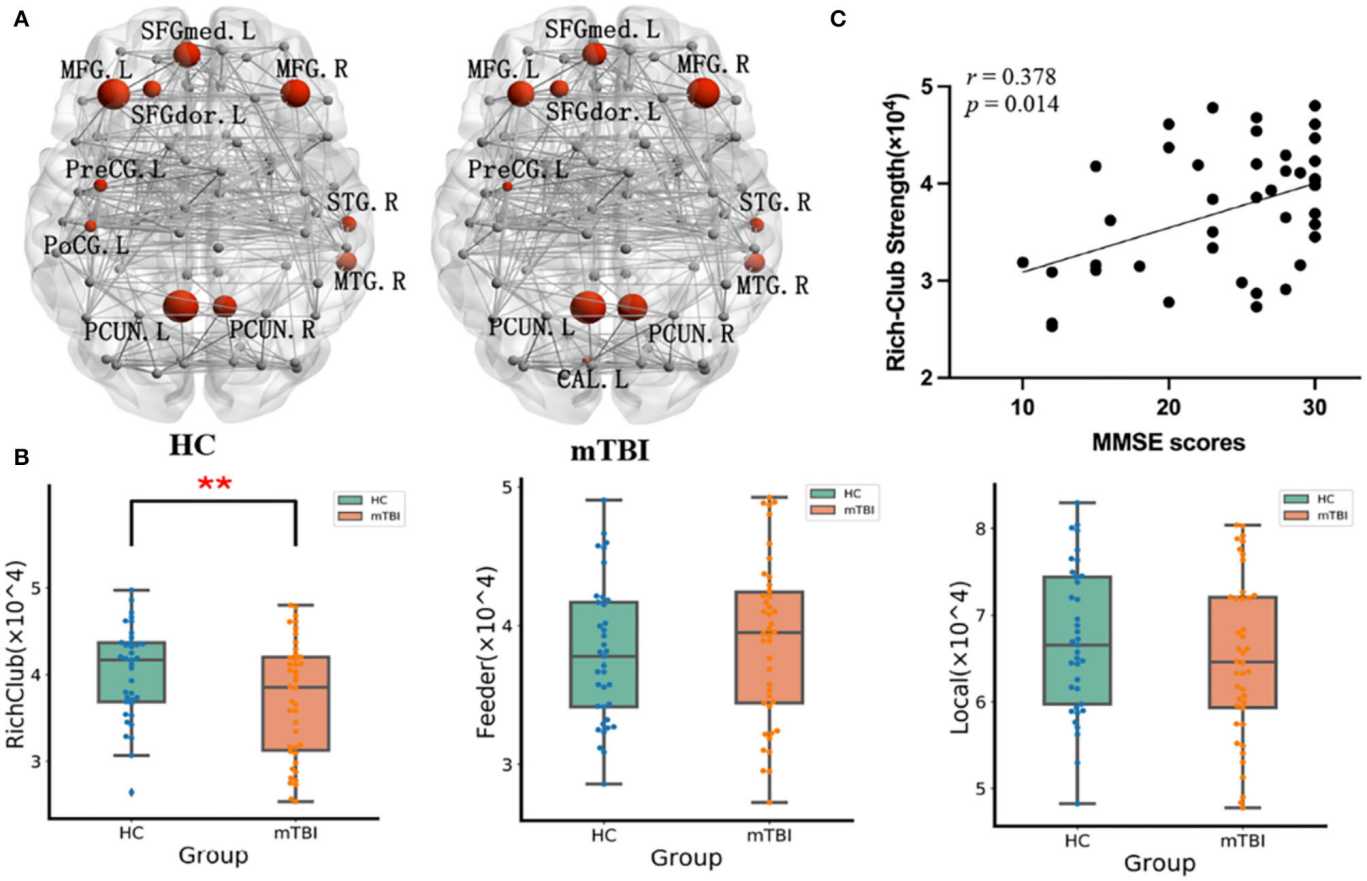
**(A)** The hubs distribution of the WM networks in the mTBI and HCs group, with hubs colored in red and the hubs sizes indicating the nodal connection strength. PCUN, precuneus; MFG, middle frontal gyrus; SFGmed, medial of superior frontal gyrus; SFGdor, dorsolateral of superior frontal gyrus; PreCG, precentral gyrus; MTG, middle temporal gyrus; STG, superior temporal gyrus; PoCG, postcentral gyrus; CAL, Calcarine fissure and surrounding cortex. **(B)** The mTBI group showed significantly lower strength of the rich-club connection. The feeder and local connections were no significant change compared with HCs. **(C)** Correlation between the MMSE score and rich-club connection strength. ** Indicates *p* < 0.05. mTBI, mild traumatic brain injury; HC, healthy control; MMSE, Mini-Mental State Examination.

The distribution of hub nodes in the two groups was similar, including the bilateral precuneus, bilateral middle frontal gyrus, left medial part of the superior frontal gyrus, left dorsolateral part of the superior frontal gyrus, left precentral gyrus, right middle temporal gyrus, and right superior temporal gyrus. Compared with the HC group, the hub of the left calcarine fissure and surrounding cortex was only found in the mTBI group, and there was no hub of the left postcentral gyrus in the mTBI group (Figure 4A). Furthermore, the rich club connection strength of the mTBI group was significantly lower (*t* = 2.25, *p* = 0.027), while the feeder and local connection strengths did not change (Figure 4B). In addition, the rich club connection strength of the mTBI group was positively correlated with the MMSE scores (*r* = 0.378, *p* = 0.014) (Figure 4C).

**Figure 5:**
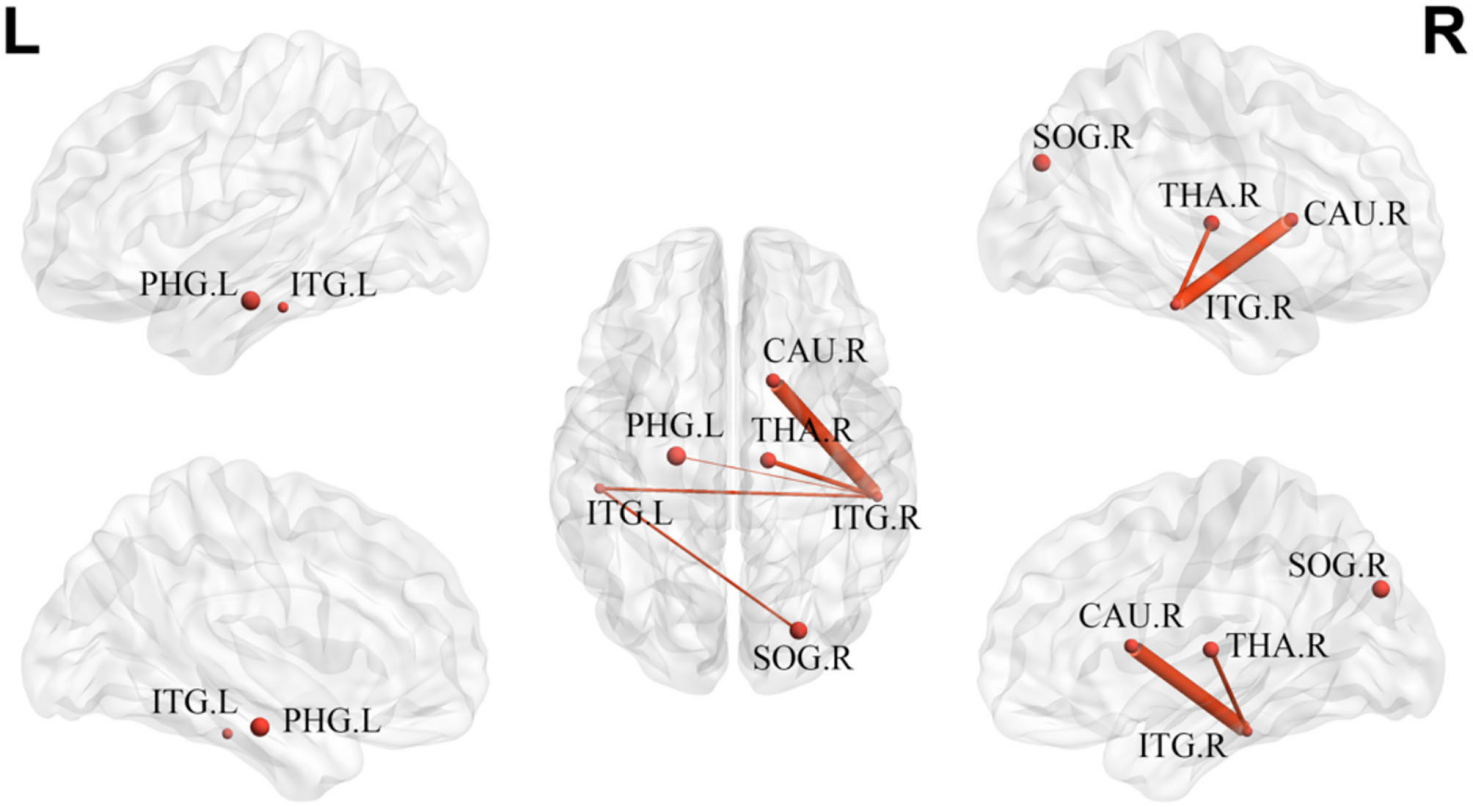
The disrupted component in mTBI group at acute stage. ITG, inferior temporal gyrus; PHG, parahippocampal gyrus; CAU, caudate nucleus; THA, thalamus; SOG, superior occipital gyrus; L, left; R, right.

Analysis of the network-based statistics showed disrupted structural connections in the mTBI group. The disrupted component was mainly distributed in the bilateral inferior temporal gyrus, left parahippocampal gyrus, right caudate nucleus, right thalamus, and right superior occipital gyrus, composed of six nodes and five edges. The thickness of the edges showed significant differences between the groups (*p* < 0.05) (Figure 5).

## Discussion

In the present study, we investigated the early microstructural changes in WM and topological changes in the brain connectome in patients with mTBI. The results showed that: (i) FA in the mTBI group increased significantly using TBSS compared with the HCs; (ii) graph theory analysis showed less nodal efficiency and increased nodal shortest path length in some areas of mTBI, mainly distributed in the right middle occipital gyrus, which was related to depressive symptoms; and (iii) the rich club connection strength of the patients with mTBI decreased and was positively correlated with the MMSE score.

Using TBSS, we found that the mTBI group in the acute phase showed significantly higher FA than the HC group, suggesting that mTBI may affect the WM integrity. Existing neuroimaging studies have demonstrated acute mTBI with high-anisotropy values (34, 35). The increased FA shortly after injury may be due to secondary injury (ischemia, cerebral hypoxia, and cerebral edema) or compensatory mechanisms (36), which cause cytotoxic swelling. Cytotoxic swelling leads to the excess of sodium ions and intracellular water accumulation. Thus, the cellular swelling narrows the free space between the neighboring axons (37), limiting the uniformity of diffusion and increasing anisotropy. A study showed that FA in patients with mTBI increases even 6 months after injury. These long-term changes may be due to the prolongation of subtle cytotoxic edema, which is more common in the developing brain (38). mTBI can lead to continuous changes in WM tracts. Usually, the WM tracts usually connect or project to the frontal lobe, including the inferior longitudinal fasciculus, inferior frontal-occipital fasciculus, and forceps minor, related to prognosis (8, 34, 39). The high FA values of the inferior frontal-occipital fasciculus and uncinate fasciculus found in the current study are consistent with the results of these previous studies. Interestingly, our study showed that the FA of WM tracts in the left hemisphere is significantly higher than that in the right side, indicating the left hemisphere is more sensitive to concussion, which is consistent with the report of DTI on the left side of football players (39). In the normal population, both DTI and post-mortem studies showed a leftward asymmetry of the WM fiber tracts in the left hemisphere regardless of subject handedness (40, 41), due to more thickly myelinated axons (42). As a result, the leftward lateralization of increased WM structure may be more susceptible to injury from the translational and rotational forces acting in concussions (43), which need further study for clinical application (44).

Global network theory analyses showed that the hierarchical structure of the mTBI group had increased. Hierarchical topology in complex networks reveals an organizational structure in which scale-free properties are combined with a high degree of clustering (45). Hierarchical topology analyses revealed that the patients with mTBI in the acute stage tended to have a more inherent association between connectivity and clustering. Previous theoretical graph theory analysis of children with acute mTBI showed significantly higher small-world properties, clustering coefficients, characteristic path length and modularity, and lower global efficiency (46). Although we found a similar trend of global network properties in the mTBI group, no significant between-group differences were found since mTBI had little effect on adults. Due to the ongoing developmental process, young patients with mTBI may be more vulnerable to brain damage than adults (47).

Compared with the HCs, the mTBI group had reduced nodal degree centrality and nodal efficiency and increased nodal shortest path length, mainly, in the bilateral temporal lobe, right middle occipital gyrus, right insula, and putamen. The changed local network metrics are likely due to disrupted global WM (axonal) integrity, although we did not find regions with changed local network properties corresponding to the regions of increased FA in patients with mTBI. The temporal lobe, located in the middle fossa of the skull, is one of the most vulnerable brain regions to injury due to cerebral trauma (48). Structural changes in the temporal lobe of the GM and WM have consistently been reported (49, 50). Our study results of decreased nodal degree centrality and nodal efficiency of the structural subnetwork in temporal regions are consistent with a study that found lower eigenvector centrality within the left temporal pole (24). As part of the salience network, the insular cortex has extensive structural connections with the prefrontal, parietal, and central cingulate gyri. It is a critical brain region for integrating processes and controlling cognitive, emotional, and behavioral functions (47). Several studies have reported the insular changes associated with mTBI (51, 52). The occipital lobe contains most of the anatomical areas responsible for visual processing and integration, contributes to communicating visual information and other sensory system information with the cerebral cortex, and plays a role in facial emotion perception (53). We found a correlation between the changed node metrics of the right middle occipital gyrus and the SDS scores in patients with mTBI. Through magnetoencephalography source analysis of untreated patients with depression, one study found that the focal magnetic low-frequency activity in the right occipital lobe was abnormal, and the increased occipital lobe delta dipole density correlated with disease severity (54). The middle occipital gyrus may be involved in the processing of cognitive bias in depression through its connection to the limbic cortex. Changes in the right middle occipital gyrus in acute mTBI may indicate potential structural brain recovery when rapid neurophysiological changes occur.

In this study, both hub and non-hub regions displayed decreased efficiency. Our study found no difference in the distribution of hubs, suggesting that acute mTBI has no significant effect on hub regions, which is consistent with the results of a previous study (46). In addition, our study showed that the strength of the rich club connection in the mTBI group was significantly lower and associated with lower cognitive performance levels. Rich club networks usually consist of highly myelinated FA fiber tracts for effective action potentials and information transmission (55). The high rotational acceleration forces can cause brain damage and changes in fiber bundles, which lead to the deformation of WM fiber bundles widely distributed throughout the brain and increase the maximum principal strains in high-fiber directionality regions compared with regions with lower fiber directionality (56). Rich club constitutes the backbone of a network with highly connected central hubs known to be vulnerable targets susceptible to disturbances in brain trauma and fundamental to high-order cognitive processes (57). One study revealed significantly decreased rich club organization in young patients with chronic TBI, which was related to impaired executive function (58). Our research shows that the subnetworks of rich clubs have changed to more mild damage, which is consistent with the results of other studies (21). As the only sufficiently sensitive subnetwork, the rich club response to mTBI may provide new evidence for the underlying mechanisms of the acute cognitive deficits.

Network-based statistics revealed a disrupted component in the mTBI group, mainly distributed in the bilateral inferior temporal gyrus, left parahippocampal gyrus, right caudate nucleus, right thalamus, and right superior occipital gyrus, including some long- and short-range connections. These results confirmed the overall disruption of structural connectivity in patients with mTBI, but to a lesser extent. The decline in component connection strength is consistent with our DTI findings that WM abnormalities are widespread in patients with mTBI during the acute phase.

Our study has several limitations. First, the degree of injury was relatively minor (the average Glasgow Coma Score was 14.5) making it difficult to overcome the inter-subject variance. We did not observe significant differences in global network properties in the adult mTBI at the acute stage compared with the HCs, which is similar to a previous study with few positive MRI findings in the mTBI group (59). Second, this study is limited by its cross-sectional design. MRI was performed at a single time point within seven days after the injury. The longitudinal changes in brain structure and the relationship between theoretical graph measures of the structural connectome and improved cognitive/emotional recovery are unclear. Finally, we did not use sufficient standard neuropsychological tests in our current study, which may provide additional factors after being affected by mTBI. This requires a more extensive and detailed description of the neuropsychological changes involved after mTBI and how they map to different injury patterns in the structural connectome. Large-scale longitudinal follow-up studies with complex standard neuropsychological tests will be conducted in the future to identify brain changes in patients with mTBI and precisely predict head trauma outcomes using machine learning methods. Achieving this goal will be the focus of future research.

## Conclusion

Using TBSS and graph theory analysis, this study found an altered WM structure and disrupted the topological organization of WM networks in mTBI at the acute stage. Re-modularization of the right occipital gyrus associated with mTBI may be linked to depressive symptoms after mTBI. Furthermore, the strength of the rich club connection may provide insight into cognitive decline in acute mTBI.

## Data Availability Statement

The raw data supporting the conclusions of this article will be made available by the authors, without undue reservation.

## Ethics Statement

The studies involving human participants were reviewed and approved by Lanzhou University Second Hospital Ethics Committee. The patients/participants provided their written informed consent to participate in this study.

## Author Contributions

WeH designed and wrote the original draft. WaH and PZ contributed to the data curation and analysis. JW and LM completed the neuropsychological assessment and collected the data. YJ and YZ conceived and revised the manuscript. JZ gave conception and funding acquisition. All authors contributed to the article and approved the submitted version.

## Funding

This work was supported by the National Natural Science Foundation of China (No. 81960309) and the Science and Technology Project of Gansu Province (No. 21JR7RA403).

## Conflict of Interest

The authors declare that the research was conducted in the absence of any commercial or financial relationships that could be construed as a potential conflict of interest.

## Publisher's Note

All claims expressed in this article are solely those of the authors and do not necessarily represent those of their affiliated organizations, or those of the publisher, the editors and the reviewers. Any product that may be evaluated in this article, or claim that may be made by its manufacturer, is not guaranteed or endorsed by the publisher.
